# Comparison of Hemorrhagic Risk between Prasugrel and Clopidogrel: a Retrospective Study using Adverse Drug Event Reporting Databases

**DOI:** 10.7150/ijms.43168

**Published:** 2020-03-05

**Authors:** Hiromi Hagiwara, Hidekatsu Fukuta, Takahiro Niimura, Yoshito Zamami, Keisuke Ishizawa, Kazunori Kimura, Takeshi Kamiya, Nobuyuki Ohte

**Affiliations:** 1 Department of Medical Innovation, Nagoya City University Graduate School of Medical Sciences.; 2 Clinical Research Management Center, Nagoya City University Hospital.; 3 Core Laboratory, Nagoya City University Graduate School of Medical Sciences.; 4 Department of Clinical Pharmacology and Therapeutics, Tokushima University Graduate School of Biomedical Sciences.; 5 Department of Pharmacy, Tokushima University Hospital.; 6 Department of Clinical Pharmaceutics, Nagoya City University Graduate School of Medical Sciences.; 7 Department of Hospital Pharmacy, Nagoya City University Graduate School of Pharmaceutical Sciences.; 8 Department of Pharmacy, Nagoya City University Hospital.; 9 Department of Cardiology, Nagoya City University Graduate School of Medical Sciences.

**Keywords:** Clopidogrel, FDA Adverse Event Reporting System, Hemorrhagic risk, Japanese Adverse Drug Event Report, Prasugrel

## Abstract

**Background**: Prasugrel inhibits platelet aggregation more potently and exerts therapeutic action faster than clopidogrel. In the global phase III trial conducted in Western and South American countries that excluded Asian countries, prasugrel reduced ischemic events but increased hemorrhagic risk compared with clopidogrel in patients with acute coronary syndrome scheduled for percutaneous coronary intervention. In the Japanese phase III trial for similar patients, the efficacy of prasugrel compared with clopidogrel was comparable to the global trial, but the safety could not be confirmed because of an insufficient number of patients. Furthermore, given the strict enrollment criteria, the results of these trials may not be applicable to routine clinical practice. Accordingly, we compared the hemorrhagic risk of prasugrel and clopidogrel in real-world settings by analyzing adverse drug event reports in post-marketing stages provided by the Japanese regulatory authorities and the U.S. Food and Drug Administration (FDA).

**Methods**: We analyzed a total of 3,970 reports for prasugrel (n = 518) or clopidogrel (n = 3,452) between 2014 and 2017 in the Japanese Adverse Drug Event Report (JADER) and a total of 91,914 reports for either prasugrel (n = 5,992) or clopidogrel (n = 85,922) between 2009 and 2019 in the FDA Adverse Event Reporting System (FAERS).

**Results**: In JADER and FAERS, prasugrel was more frequently and significantly associated with hemorrhagic event reports than clopidogrel. After adjustment for known confounders including age, sex, and concomitant medications (aspirin, anticoagulants, and proton pump inhibitors), the hemorrhagic risk of prasugrel compared with clopidogrel remained significant (adjusted reporting odds ratios [95% CI] for total, intracranial, and gastrointestinal hemorrhagic events = 2.42 [1.97-2.96], 2.45 [1.85-3.24], and 2.27 [1.73-2.97] in JADER, and 2.21 [2.09-2.34], 1.21 [1.09-1.33], and 1.41 [1.29-1.54] in FAERS).

**Conclusions**: The hemorrhagic risk was found to be greater with prasugrel than clopidogrel in real-world patients, including Japanese patients.

## Introduction

Clopidogrel and prasugrel are P2Y12 inhibitors, thienopyridine antiplatelet agents. Prasugrel is a new-generation agent that inhibits platelet aggregation more potently and exerts therapeutic action faster than clopidogrel 1,2. Clopidogrel and prasugrel show markedly different pharmacokinetic and pharmacodynamic profiles. Clopidogrel is a prodrug bio-transformed into its active moiety by cytochrome P450 (CYP) 2C19, while prasugrel is bio-transformed by CYP 3A4 and CYP 2B6. Because of the higher prevalence of CYP 2C19 loss-of-function alleles, a decreased response to clopidogrel is more common in Asian patients than in non-Asian patients.

In the global phase III trial (TRITON-TIMI38), which was conducted in 30 Western and South American countries, but no Asian countries, prasugrel reduced ischemic events but increased hemorrhagic risk compared to clopidogrel in patients with acute coronary syndromes (ACS) with a scheduled percutaneous coronary intervention (PCI) 3. In the Japanese phase III trial (PRASFIT-ACS) for similar patients, the efficacy of prasugrel compared to clopidogrel was comparable to the global trial, but the safety could not be confirmed due to an insufficient number of patients 4. Furthermore, due to the strict enrollment criteria, the patients who participated in these trials might represent a select group of patients, and thus, the results of these trials may not be applied to routine clinical practice.

Accordingly, the purpose of the present study was to compare the hemorrhagic risk of prasugrel and clopidogrel in real-world settings, including Japan. We analyzed adverse event report databases at post-marketing stages, which were provided by Japanese regulatory authorities and the U.S. Food and Drug Administration (FDA). Adverse event report databases are valuable tools used in post-marketing surveillance that reflect the realities of clinical practice 5.

## Methods

The Japanese Adverse Drug Event Report (JADER) and the FDA Adverse Event Reporting System (FAERS) include several million spontaneous reports of drug-associated adverse events from healthcare professionals and pharmaceutical companies 6. In Japan, JADER was established by the Pharmaceuticals and Medical Devices Agency (PMDA) 7. JADER includes reports only from Japanese sources. FAERS includes reports from US sources; serious and unlabeled spontaneous reports from non-US sources including Asian countries etc.; and serious, unlabeled and attributable post-marketing clinical trial data. For this analysis, adverse event reports were downloaded from the PMDA and FDA websites. We used JADER data from the second quarter (Q2) of 2014 to 2017 Q2, which are publicly available on the PMDA website. For FAERS analysis, data from 2009 Q2 to the first quarter (Q1) of 2019, which are publicly available on the FDA website, were used. The downloaded data were processed using Microsoft Access 2016® (Microsoft, Redmond, WA).

In the JADER and FAERS databases, adverse events are coded according to the terminology preferred by the Medical Dictionary for Regulatory Activities (MedDRA, http://www.meddra.org/; version 22.0). The Standardized MedDRA Query (SMQ) was used for hemorrhagic events (SMQ code: 20000038(39-40)), intracranial (IC) hemorrhagic events (SMQ code: 20000064), and gastrointestinal (GI) hemorrhagic events (SMQ code: 20000108). All reported adverse events of interest (“hemorrhagic events”) were defined as “cases” and all reported other adverse events as “non-cases.” Drugs in the FAERS database are registered arbitrarily; for example, they may be registered as generic or brand names, or as abbreviations. Drug Bank (version 5.1, The Metabolomics Innovation Centre, Canada, http://www.drugbank.ca/) is a reliable drug database used as a reference in pharmacovigilance analyses. Therefore, we used Drug Bank as a source for batch conversion and compilation of drug names. The drugs selected for this investigation were clopidogrel, prasugrel, proton pump inhibitors (PPIs) (omeprazole, esomeprazole, lansoprazole, rabeprazole, pantoprazole, and vonoprazan fumarate), anticoagulants (warfarin, dabigatran, rivaroxaban, apixaban, and edoxaban) and aspirin. Before analyzing the data, a text-mining approach was used that stated the drugs in terms of their generic names.

Differences in categorical data at baseline between groups in JADER and FAERS were compared by the Fisher exact probability test. Reporting odds ratios (RORs) were used for evaluating signal detection as previously reported 7. Adjusted RORs were calculated using multivariate logistic regression to control for age, sex, and concomitant medications that could have affected the hemorrhagic risk. Statistical significance was defined as a P-value <0.05. Analyses were performed with the use of R software, version 3.4.3 (R Development Core Team, 2009).

## Results

**Figure 1:**
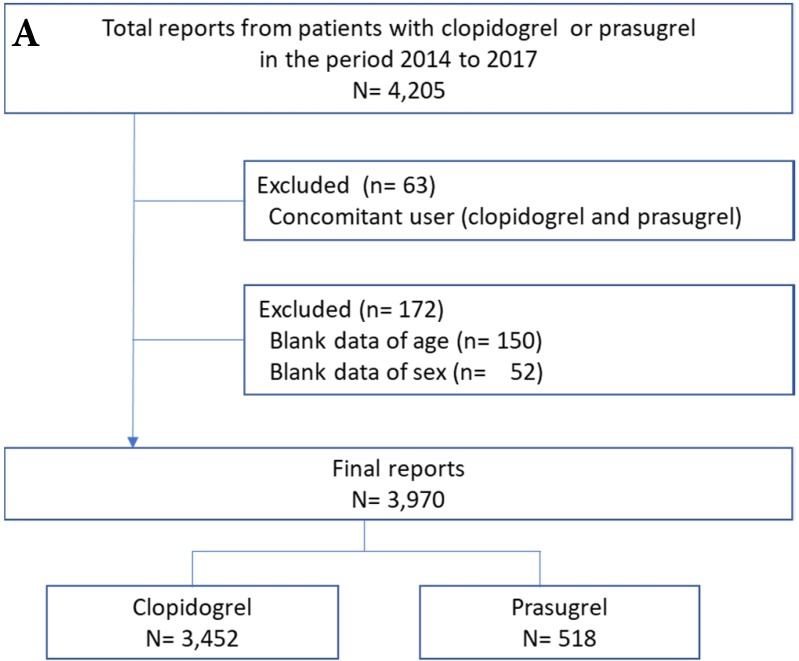

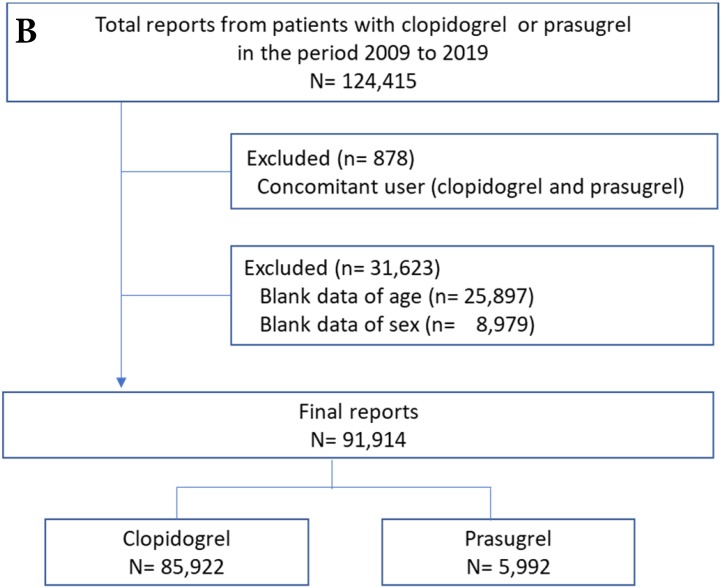
Flowchart of the JADER **(A)** and FAERS **(B)** report. JADER and FAERS indicates the Japanese Adverse Drug Event Report and the FDA Adverse Event Reporting System, respectively.

Adverse event reports of prasugrel and clopidogrel groups are shown in Figure 1. After exclusion of the reports due to the concomitant use of clopidogrel and prasugrel and the blank data of age and sex (6% for JADER and 26% for FAERS), the final number of reports for analysis was 3,970 for JADER and 91,914 for FAERS.

The number of hemorrhagic event reports is shown in Table 1. Hemorrhagic events were generally more frequently reported in the prasugrel group than in the clopidogrel group in JADER and FAERS.

Clinical characteristics of study patients are shown in Table 2. Compared with the clopidogrel group, the prasugrel group was more likely to use aspirin and PPIs in JADER. The prasugrel group was more likely to include mostly males and be using aspirin compared with the clopidogrel group in the FAERS.

The hemorrhagic risk of prasugrel compared with clopidogrel in JADER and FAERS is shown in Table 3. Prasugrel had a greater and significant association with hemorrhagic event reports compared with clopidogrel in JADER (unadjusted ROR [95% CI] for total, IC, and GI hemorrhagic events = 2.91 [2.41-3.52], 2.58 [1.99-3.33], and 2.25 [1.76-2.87], respectively). Similar results were observed in FAERS (unadjusted ROR [95% CI] for total, IC, and GI hemorrhagic events = 1.72 [1.63-1.82], 1.18 [1.06-1.30], and 1.09 [1.00-1.18], respectively). After adjustment for known confounders including age, sex, and concomitant medications, hemorrhagic risk of prasugrel compared to clopidogrel remained significant (adjusted ROR [95% CI] for total, IC, and GI hemorrhagic events = 2.42 [1.97-2.96], 2.45 [1.85-3.24], and 2.27 [1.73-2.97] in JADER and 2.21 [2.09-2.34], 1.21 [1.09-1.33], and 1.41 [1.29-1.54] in FAERS, respectively).

## Discussion

The purpose of the present study was to compare the hemorrhagic risk of prasugrel and clopidogrel in real-world settings, including Japan. We observed that prasugrel had a more frequent and significant association with hemorrhagic events than clopidogrel in both JADER and FAERS databases. These observations suggest that prasugrel may have a greater hemorrhagic risk than clopidogrel in real-world patients. To the best of our knowledge, the present study is the first to compare the hemorrhagic risk of prasugrel and clopidogrel by analyzing JADER.

Consistent with the present study, two previous studies reported that prasugrel had more hemorrhagic events than clopidogrel by analyzing the FAERS database 8,9. However, we believe that these studies have several major limitations. First, these studies did not adjust for potential confounding factors. Second, the study periods in the two studies were relatively short (only 2015 8 and 2014-2016 9). The strengths of the present study include the adjustment for known confounding factors including age, sex, and concomitant medications (anticoagulants, aspirin, and PPIs) and a longer study period (2009-2019) with a larger number of reports. In the present study, we analyzed a total of 91,914 reports in the FAERS database in which patients were either treated with prasugrel (n = 5,992) or clopidogrel (n = 85,922). However, two previous studies included only approximately 3,000 reports of prasugrel and 13,200 reports of clopidogrel 8, as well as approximately 500 reports of prasugrel and 2,200 reports of clopidogrel 9. Long-term results indicate that there is a risk of hemorrhagic events without a bias that was increased reports immediately after launch. On analyzing a large number of cases, the hemorrhagic risk was noted to be high regardless of patient background. We could detect the hemorrhage risk signal from spontaneous reporting systems (SRSs) more accurately by adjusting for the confounding factors.

The present results are inconsistent with those of the two phase III trials for Japanese patients undergoing PCI, in which there was no significant difference in the incident rate of bleeding-related adverse events between the prasugrel and clopidogrel groups 4,10. However, these trials were designed primarily to assess the efficacy of prasugrel compared to clopidogrel, and thus, the difference in observation may be due to underpowered enrollment. Therefore, the present study is the first to compare the hemorrhagic risk of prasugrel and clopidogrel in a large number of Japanese patients in real-world settings.

The present study has several clinical implications. Given the observed higher hemorrhagic risk with prasugrel compared with clopidogrel, short-term treatment with prasugrel or low-dose treatment with prasugrel may be an option for patients with a particularly high hemorrhagic risk 11-13. Specifically, the TROPICAL-ACS trial compared the ischemic and bleeding event rates between standard treatment with prasugrel for 1 year and a step-down regimen (1-week prasugrel treatment followed by 1-week clopidogrel and maintenance therapy with clopidogrel or prasugrel guided by platelet function testing) in white patients with ACS undergoing coronary revascularization 11. This trial showed that both ischemic and bleeding event rates tended to be lower in a step-down regimen group than in standard treatment group. Furthermore, a prospective observational study reported that the ischemic and bleeding event rates were similar between Japanese ACS patients at high bleeding risk receiving a low-maintenance dose of prasugrel and those at non-high bleeding risk receiving a standard maintenance dose of prasugrel 12. However, given the known marked interethnic differences in the pharmacokinetic and pharmacodynamic profiles between prasugrel and clopidogrel 1,2, the findings of these studies 11,12 may not be applicable to other ethnic groups. Further studies are necessary to examine the efficacy and safety of a step-down regimen or a low-dose prasugrel regimen in various ethnic populations. The differences in safety and efficacy between Asian and Caucasians should be carefully evaluated in the future.

There are several limitations to this study. First, the results were obtained from SRSs such as the JADER and FAERS database, and thus, should be interpreted with caution. Most importantly, SRS is a passive reporting system, and is, therefore, subject to many biases, such as under-reporting and over-reporting 14. In addition, SRS is subjected to the “Weber effect,” which refers to an increase in adverse event reporting over the initial 2 years after a drug is approved, followed by a rapid decline in reporting rates 15. However, no such effect was evident in our database; the number of reports of total hemorrhage due to prasugrel in JADER was 130 (2014 Q2-2016 Q1) and 173 (2016 Q2-2017 Q2) and that in FAERS was 227 (the fourth quarter [Q4] of 2009 -the third quarter [Q3] of 2011) and 305 (2011 Q4-2013 Q3). Furthermore, owing to the longer survey period (3 years in JADER and 10 years in FAERS) in the present study, it is unlikely that the Weber effect had a substantial impact on our results. Second, despite adjustment for known confounders including age, sex, and concomitant medications, our observed higher hemorrhagic risk with prasugrel compared with clopidogrel might reflect unreported factors that are related to hemorrhagic events, such as medical history, comorbidities, the duration of antiplatelet therapy, and clinical test data. It is impossible to evaluate the “true” safety risk without baseline clinical characteristics information. In general, ROR cannot be used to infer the comparative strength of causality 5. However, it offers a rough indication of the signal strength that can be used to generate hypotheses to search for unknown potential adverse events. Using our confounding adjustment, the hemorrhagic risk signal could be more accurately evaluated than in previous studies. The unadjusted ROR value of the previous studies 8,9 is different from the unadjusted value of the present study due probably to the different study period. However, it is important to recognize that the hemorrhagic risk signal was consistently detected across the studies using SRS. Third, information regarding the indication for these drugs in JADER and FAERS databases is lacking. Prasugrel is indicated in patients with coronary artery disease, whereas clopidogrel is indicated in patients with cerebrovascular disease or peripheral arterial disease in addition to those with coronary artery disease (https://labels.fda.gov/, https://www.info.pmda.go.jp/psearch/html/menu_tenpu_base.html). Therefore, it is possible that there is a difference in clinical characteristics between patients treated with clopidogrel and those with prasugrel in our databases. However, coronary artery disease, cerebrovascular disease, and peripheral arterial disease are atherosclerotic diseases and share common risk factors. In large global registries, which include data from Japan, the clinical characteristics between patients with coronary artery disease and those with cerebrovascular disease are reportedly similar 16. Therefore, it is unlikely that the difference in the indications for clopidogrel and prasugrel has a substantial impact in our findings. Finally, the adverse events in the JADER and FAERS databases were reported according to the MedDRA, which did not permit us to evaluate the severity of hemorrhagic events.

## Conclusion

The present analysis suggests that prasugrel may have a higher hemorrhagic risk than clopidogrel in real-world patients, including Japanese patients. Given the inherent limitations of using SRSs, our findings should be confirmed in registry studies or, ideally, new well-designed studies such as registry-based randomized, controlled trials.

## Figures and Tables

**Table 1:** Adverse event profile of clopidogrel and prasugrel using the JADER and FAERS databases

	JADER					FAERS				
	Clopidogrel	%*	Prasugrel	%*	P-value	Clopidogrel	%*	Prasugrel	%*	P-value
Total reports	3,452		518			85,922		5,992		
Hemorrhagic events	1,126	32.6	303	58.5	<0.001	20,788	24.2	2,123	35.4	<0.001
IC hemorrhagic events	267	7.7	92	17.8	<0.001	5,715	6.7	463	7.7	0.001
GI hemorrhagic events	332	9.6	100	19.3	<0.001	8,973	10.4	673	11.2	0.057

FAERS indicates the FDA adverse event reporting system; GI: gastrointestinal; IC: intracranial; JADER: the Japanese adverse drug event report. *Values are percentage (within the group).

**Table 2:** Baseline clinical characteristics

	JADER					FAERS				
	Clopidogrel	%^*^	Prasugrel	%^*^	P-value	Clopidogrel	%^*^	Prasugrel	%^*^	P-value
Total reports	3,452		518			85,922		5,992		
Age>60 years	3,116	90.3	447	86.3	0.008	67,507	78.6	3,800	63.4	<0.001
Men	2,409	69.8	383	73.9	0.056	48,800	56.8	3,928	65.6	<0.001
Aspirin	1,538	44.6	476	91.9	<0.001	45,330	52.8	3,449	57.6	<0.001
Anticoagulants	498	14.4	79	15.3	0.640	12,951	15.1	483	8.1	<0.001
PPIs	1,346	39.0	329	63.5	<0.001	7,379	8.6	486	8.1	0.210

FAERS indicates the FDA adverse event reporting system; JADER, the Japanese adverse drug event report; PPIs, proton pump inhibitors. *Values are percentage (within the group).

**Table 3:** Hemorrhagic risk with prasugrel compared with clopidogrel in JADER and FAERS

	JADER				FAERS			
	Unadjusted ROR (95% Cl)	P-value	Adjusted ROR (95% Cl)	P-value	Unadjusted ROR (95% Cl)	P-value	Adjusted ROR (95% Cl)	P-value
**Total hemorrhagic risk**								
Prasugrel vs. clopidogrel	2.91 (2.41-3.52)	< 0.001	2.42 (1.97-2.96)	< 0.001	1.72 (1.63-1.82)	< 0.001	2.21 (2.09-2.34)	< 0.001
Age >60 years	1.09 (0.88-1.36)	0.414	1.22 (0.97-1.52)	0.088	1.45 (1.40-1.51)	< 0.001	1.39 (1.33-1.44)	< 0.001
Men	0.90 (0.78-1.03)	0.129	0.84 (0.73-0.98)	0.024	1.14 (1.11-1.18)	< 0.001	1.00 (0.97-1.04)	0.792
Aspirin	1.91 (1.67-2.18)	< 0.001	1.64 (1.43-1.90)	< 0.001	1.69 (1.64-1.74)	< 0.001	1.65 (1.60-1.71)	< 0.001
Anticoagulants	2.50 (2.09-2.99)	< 0.001	2.61 (2.17-3.14)	< 0.001	6.57 (6.32-6.83)	< 0.001	6.56 (6.31-6.83)	< 0.001
**IC hemorrhagic risk**								
Prasugrel vs. clopidogrel	2.58 (1.99-3.33)	< 0.001	2.45 (1.85-3.24)	< 0.001	1.18 (1.06-1.30)	0.001	1.21 (1.09-1.33)	< 0.001
Age >60 years	1.10 (0.76-1.59)	0.609	1.16 (0.80-1.69)	0.432	0.84 (0.79-0.89)	< 0.001	0.81 (0.76-0.86)	< 0.001
Men	1.04 (0.82-1.32)	0.760	1.00 (0.79-1.28)	0.972	0.94 (0.90-0.99)	0.031	0.90 (0.86-0.95)	< 0.001
Aspirin	1.43 (1.15-1.78)	0.001	1.14 (0.90-1.45)	0.284	1.15 (1.09-1.21)	< 0.001	1.11 (1.05-1.17)	< 0.001
Anticoagulants	2.22 (1.72-2.87)	< 0.001	2.23 (1.72-2.89)	< 0.001	1.82 (1.71-1.94)	< 0.001	1.88 (1.76-2.00)	< 0.001
**GI hemorrhagic risk**								
Prasugrel vs. clopidogrel	2.25 (1.76-2.87)	< 0.001	2.27 (1.73-2.97)	< 0.001	1.09 (1.00-1.18)	0.054	1.41 (1.29-1.54)	< 0.001
Age >60 years	1.88 (1.25-2.84)	0.002	2.01 (1.33-3.05)	< 0.001	1.73 (1.63-1.83)	< 0.001	1.57 (1.47-1.67)	< 0.001
Men	1.10 (0.88-1.37)	0.423	1.05 (0.83-1.31)	0.698	1.27 (1.21-1.32)	< 0.001	1.09 (1.05-1.15)	< 0.001
Aspirin	1.62 (1.32-1.99)	< 0.001	1.52 (1.22-1.90)	< 0.001	2.10 (2.01-2.20)	< 0.001	2.03 (1.94-2.13)	< 0.001
Anticoagulants	1.82 (1.42-2.32)	< 0.001	1.83 (1.42-2.35)	< 0.001	7.44 (7.10-7.78)	< 0.001	7.07 (6.75-7.41)	< 0.001
PPIs	0.61 (0.49-0.76)	< 0.001	0.50 (0.40-0.63)	< 0.001	0.82 (0.76-0.89)	< 0.001	0.76 (0.70-0.83)	< 0.001

Cl indicates confidence interval; FAERS: the FDA adverse event reporting system; GI: gastrointestinal; IC: intracranial; JADER: the Japanese adverse drug event report; PPIs: proton pump inhibitors; ROR: reporting odds ratios.
